# Buried Autologous Breast Reconstruction: Outcomes and Technical Considerations

**DOI:** 10.3390/jcm13051463

**Published:** 2024-03-02

**Authors:** Henrietta Creasy, Isabelle Citron, Timothy P. Davis, Lilli Cooper, Asmat H. Din, Victoria Rose

**Affiliations:** Plastic Surgery Department, St Thomas’ Hospital, Westminster Bridge Road, London SE1 7EH, UKtimothy.davis@gstt.nhs.uk (T.P.D.);

**Keywords:** single stage, buried, venous flow coupler, autologous, breast reconstruction

## Abstract

The purpose of this study is to compare outcomes in patients undergoing buried and non-buried free flaps for breast reconstruction, in addition to evaluating the safety and reliability of venous flow couplers. A retrospective review was performed of all patients undergoing free flap breast reconstruction between 2013 and 2023. The primary outcomes were free flap failure, complications and the number of procedures required to complete the reconstructive journey. A total of 322 flaps were performed in 254 consecutive patients, with 47.5% (*n* = 153) being buried and 52.0% (*n* = 169) being non-buried reconstructions. The most common flap of choice being deep inferior epigastric artery perforator flaps (81.9%) followed by profunda artery perforator flaps (14.3%). There was no significant difference between the two groups in complications, including flap failure (buried 2.0% vs. non-buried 1.8% *p* = 0.902). There was a significant reduction in the number of procedures required to complete the reconstructive journey, with 52.2% (*n* = 59) of patients undergoing single-stage breast reconstruction in the buried group compared with only 25.5% (*n* = 36) in the non-buried group (*p* < 0.001). Two (0.6%) patients experienced a false negative in which the signal of the flow coupler was lost but the flap was perfused during re-exploration. No flap losses occurred without being identified in advance by a loss of audible venous flow signal. Buried free flap breast reconstruction is safe and requires fewer operations to complete patients’ reconstructive journey. Flow couplers are a safe and effective method of monitoring buried free flaps in breast reconstruction.

## 1. Introduction

Autologous breast reconstruction is the gold standard following mastectomy for breast cancer [1]. In a delayed setting, a skin paddle is required to address the deficiency of skin. By contrast, in an immediate setting, skin sparing mastectomy classically involves a skin paddle at the site of the excised nipple areolar complex (NAC). With an increasing move towards nipple sparing mastectomies (NSM), the requirement for a skin paddle is often solely for the clinical monitoring of flap perfusion [2,3,4]. Adopting alternative methods of flap monitoring enables buried flap reconstruction, with de-epithelisation of the entire flap and preservation of the native breast envelope [5].

Combining NAC preservation with buried flaps can significantly reduce the stigmata of surgery on the reconstructed breast. This has been shown to improve sexual and psychological wellbeing, reducing the number of symmetrising and adjustment procedures required and thus streamlining the patient journey and avoiding “reconstructive burnout” [6,7,8,9]. However, this is yet to become standard practice in most specialist centres. 

While an early series of buried flaps raised concerns about the reliability of monitoring and a high revision rate [10], more recently published data has demonstrated their safety [11]. Our recently published series demonstrated buried flaps to be safe and reliable, with a comparable flap failure rate and fewer revisions over time than a matched cohort of flaps with a skin paddle [6]. A total of 50% patients with buried flaps had single-stage reconstruction compared to 29% in the skin paddle group. This was due to the preservation of the native NAC negating the requirement for nipple reconstruction, in combination with fewer secondary adjustments of the breast mound with lipofilling or liposuction or the need to excise the monitoring skin paddle. 

Despite evidence of the safety of buried flaps, one limiting factor to consider is a lack of familiarity with venous flow couplers used to monitor the flaps. Most of the literature surrounding their use is within the field of head and neck reconstruction [12]. Whilst some small studies exist, there remains a lack of evidence regarding their positive predictive value of identifying a compromised flaps in breast reconstruction [13]. Others have questioned the potential morbidity associated with their removal, with a perceived higher rate of haematoma and flap loss [14].

This study aims to compare the outcomes of patients undergoing autologous breast reconstruction using buried and non-buried flaps. The primary outcomes were flap loss, the number of procedures required to complete the reconstructive journey and complications. In addition, we will also explore the reliability and accuracy of flow couplers in breast flap monitoring and present a learning curve associated with their use. To the author’s knowledge, this will constitute the largest published series of buried flaps for breast reconstruction.

## 2. Methods

### 2.1. Subjects

All patients undergoing autologous breast reconstruction by the senior author (VR) between 2013 and August 2023 were included for review. This included patients undergoing reconstruction at three units in the public and private sectors in London, UK. Demographics, indications, pre-operative, intra-operative and post-operative details; complications; and further surgeries to improve breast appearance were collected. All data were collected from a prospectively maintained database or from the patients’ electronic record. Flap failure was defined as an entire flap that was unsalvageable following re-exploration. Partial flap failure was a flap that underwent subsequent sub-total debridement secondary to poor perfusion. Fat necrosis was defined clinically as palpable induration with or without further intervention. 

### 2.2. Pre-Operative

Pre-operatively, all patients were offered implant-based and autologous-based breast reconstruction. The requirement for adjuvant radiotherapy was not a contraindication for immediate autologous reconstruction. Patients were shown representative photographs of flap reconstructions from deep inferior epigastric perforator (DIEP), profunda artery perforator (PAP) and superior gluteal artery perforator (SGAP) flaps, and if all were feasible, they were offered a choice of reconstruction. All patients who were deemed oncologically safe by the multidisciplinary team were offered NSM. Patients were offered simultaneous symmetrisation using mastopexy, reduction mastopexy or fat grafting where it was deemed that this was likely to be necessary at any point in the future. 

### 2.3. Intra-Operative

#### Skin Envelope

**Figure 1:**
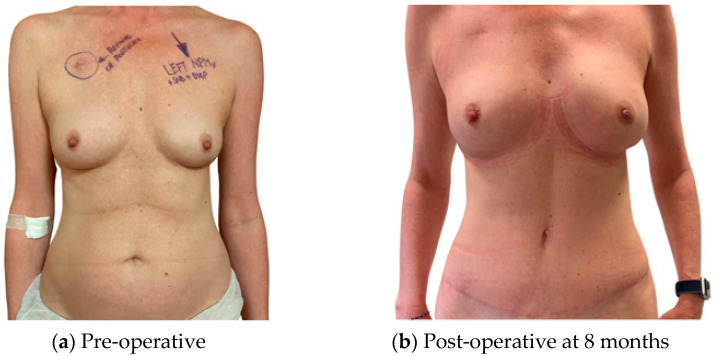
Left NSM with an IMF incision; immediate buried DIEP.

**Figure 2:**
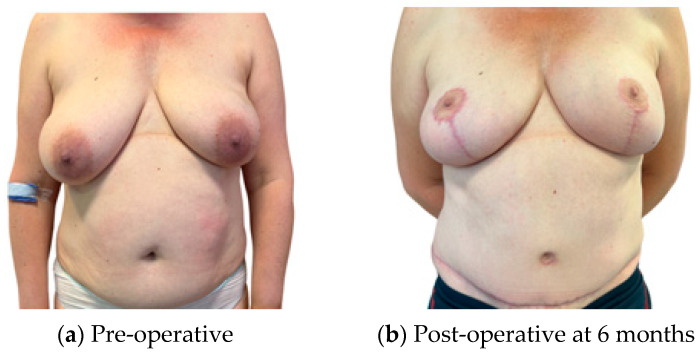
Bilateral NSM with Wise pattern skin excision; nipple preserved on inferior dermal pedicle; immediate buried DIEP.

**Figure 3:**
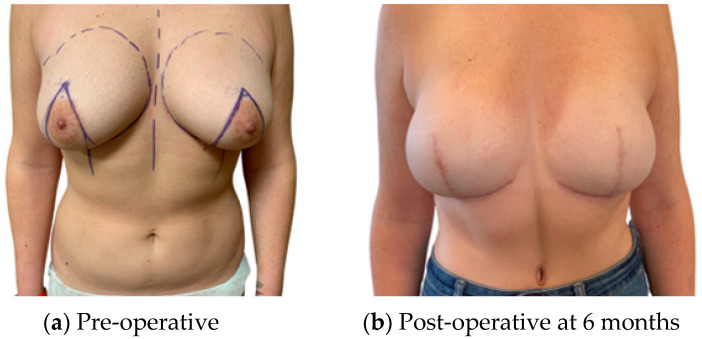
Bilateral skin reducing mastectomy with Wise pattern excision; immediate buried DIEP flap.

**Figure 4:**
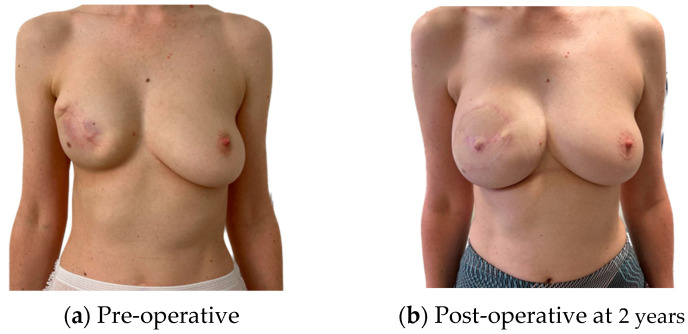
Delayed reconstruction of right breast, removal of implant and exchange for stacked PAPs with skin paddle due to inadequate skin envelope; subsequent lipofilling and nipple reconstruction with local flap.

**Figure 5:**
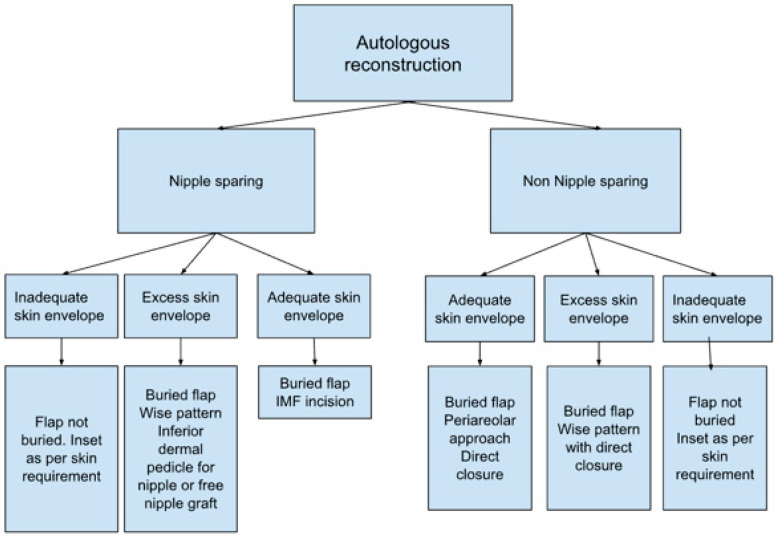
Our algorithm for the approach to mastectomy and flap inset.

For patients satisfied with their pre-operative shape, an infra mammary fold (IMF) incision for the mastectomy and a buried flap were preferred, as this gives the least surgical stigmata in patients undergoing NSM (Figure 1). In patients requiring management of the skin envelope due to ptosis or a reduction in volume, the preferred mastectomy approach was a Wise pattern, with a buried flap, where the nipple was preserved on an inferior dermal pedicle or replaced as a free nipple graft (Figure 2). For patients in which the nipple could not be preserved for oncological reasons, but the skin envelope was adequate, a periareolar approach was taken and directly closed post-operatively. In those in which the nipple was sacrificed but the skin envelope required management, the Wise pattern was closed entirely to bury the flap (Figure 3). In cases of delayed reconstruction or where a significant volume increase occurred, the additional skin was provided by the flap and the flap was not buried (Figure 4). The algorithm for the approach to mastectomy and flap inset is summarised in Figure 5. 

Symmetrisation was usually undertaken as a Wise pattern mastopexy or reduction. Where the flap was larger than the initial breast, immediate fat grafting to the contralateral breast was performed.

DIEP, PAP and SGAP flaps were raised in a standard fashion and anastomosed to the internal mammary artery (IMA) and internal mammary vein (IMV) at the level of the 3rd or 4th intercostal. Where flaps were stacked, they were anastomosed most commonly with an antegrade/retrograde approach onto the IMA/IMV or onto a large side branch for an intra-flap anastomosis. When performed, the intra-flap anastomosis was most commonly performed using a flow coupler for arterial and venous anastomosis.

**Figure 6:**
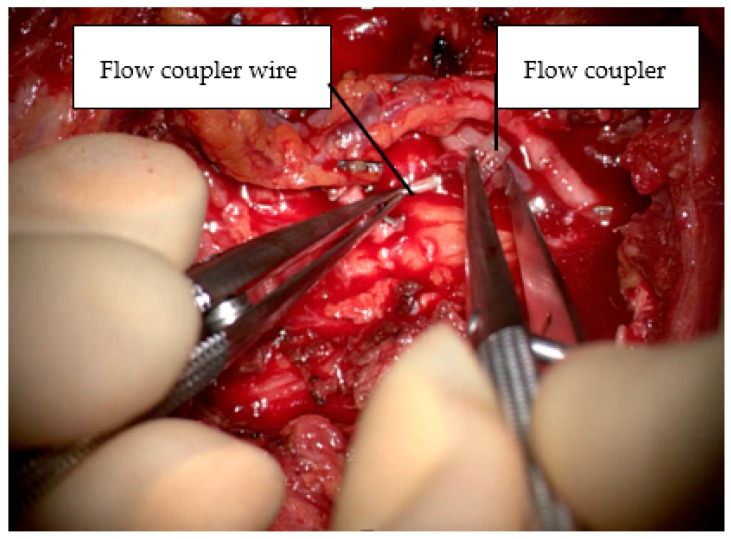
Reinsertion of flow coupler wire into the dock post-anastomosis.

**Figure 7:**
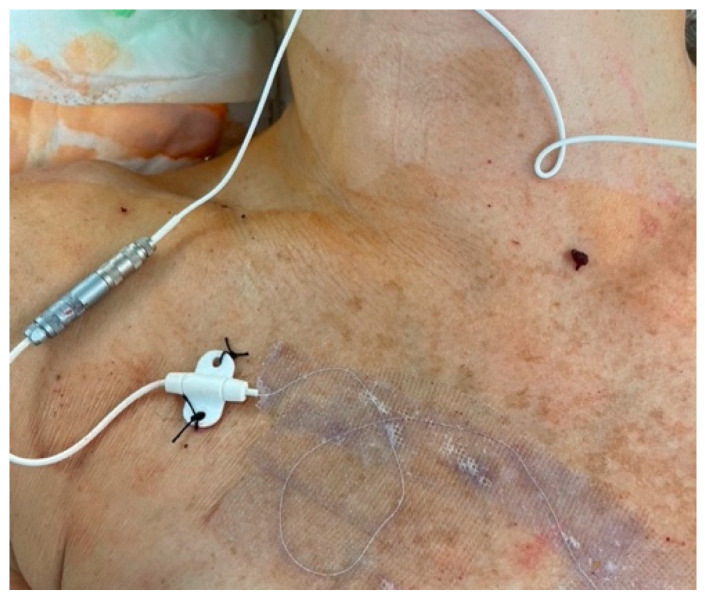
External coupler wire secured with mesh/glue Prineo™.

Flow couplers (Synovis™, Birmingham, AL, USA) were used to monitor flow at all venous anastomotic sites. When placing the coupler, the wire is removed from the dock before a standard venous coupler anastomosis is performed. A 16G cannula is then passed “in to out” such that the wire can be passed “out to in” prior to reinserting the wire into the dock (Figure 6). The external wire is secured to the skin with Dermabond™ Prineo™ (Ethicon, Somerville, NJ, USA) to reduce the risk of accidental displacement. Care should be taken to disconnect wires when removing drapes, fitting the bra and transferring the patient from the operating table. All wounds were closed with STRATAFIX^TM^ (Ethicon, Somerville, NJ, USA) and Dermabond™ Prineo™ skin closure system (Figure 7).

### 2.4. Post-Operative

Post-operatively, patients with buried flaps were monitored using flow couplers, and they were monitored for excessive swelling, firmness and high drain output. For patients with visible skin paddles, standard monitoring for capillary refill, colour, temperature and Doppler was undertaken. Flow coupler wires were removed with gentle traction prior to discharge on post-operative day 2 or 3. Patients underwent a standard enhanced recovery program, with the avoidance of excess intra-operative fluids and minimal post-operative opiate analgesia [15].

### 2.5. Statistical Tests

Descriptive statistics were used to compare demographics, operative times, complications and the number of revisions between patients undergoing buried and non-buried flaps. A *t*-test was used for continuous variables, and a z-test of proportions was used to compare incidences between groups. A value of *p* < 0.05 was considered statistically significant. Where missing data were present, variables were averaged with the available data points.

Results are reported as per the Strengthening the Reporting of Observational studies in Epidemiology (STROBE) guidelines for case–control studies [16].

## 3. Results

### 3.1. Demographics and Flap Details

A total of 322 flaps were performed in 254 consecutive patients by the senior author (VR) between 2013 and 2023 for autologous breast reconstruction. The proportion of buried flaps was 47.5%, whilst 52.0% were not buried with a cutaneous paddle. The demographics, flap types and indications are presented in Table 1. The mean follow-up was 30.2 months (Standard Deviation (SD) ± 27.8). There were no significant differences in patient demographics or flap type selection between the groups. The most common flap choice being the DIEP flap (81.9%) followed by PAP flaps (14.3%). There were significantly more immediate reconstructions in the buried flap group and similarly a greater proportion of nipple sparing and skin-reducing procedures within this cohort. Of the patients undergoing buried flaps, 24.7% (*n* = 28) underwent immediate symmetrisation with a contralateral reduction or mastopexy. This compared to 18.4% (*n* = 26) in the non-buried group.

**Figure 8:**
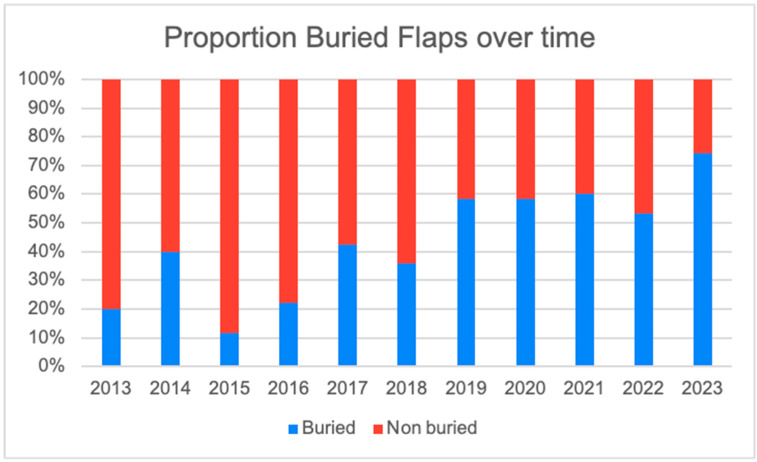
Proportion of buried vs. non-buried flaps over time.

Over time, there was a trend towards an increasing proportion of buried free flaps, with 74.3% of breast reconstructions being buried in 2023 (Figure 8).

### 3.2. Intra-Operative Results

The average ischaemia time in the buried flap group was 62.6 min (SD ± 24.7) compared to 69.0 min (SD ± 24.9) in the non-buried group (*p* = 0.036). Operating time was significantly longer in the buried cohort (buried: 416.4 min (SD ± 117.92); non-buried: 383.6 min (SD ± 287.8), *p* = 0.031). Intra-operative details are reported in Table 2.

### 3.3. Complications

There was a significantly longer follow-up period in the non-buried flap group (41.4 months (SD ± 29.2)) compared to patients undergoing buried free flap reconstruction (12.8 months (SD ± 11.4).

There were six flap losses (1.7%) and one partial flap loss (0.3%), of which two occurred in two individual patients undergoing a buried four-flap procedure. In the buried flap group, there were three (2.0%) returns to theatre in which a clinical concern with the flap was not found (a negative takeback). Two of these were due to the loss of coupler signal, and one was due to swelling. There was one negative takeback in the non-buried group due to swelling. There were no significant differences in the incidence of any complications between the groups, as seen in Table 3.

### 3.4. Additional Procedures

There was a significant difference between the two groups in the proportion of women who required additional procedures to improve the appearance of their breasts. The majority (52.2%, *n* = 59) of women in the buried group did not require further surgery to improve their breast appearance compared to 25.5% (*n* = 36) of those in the non-buried group (*p* < 0.001). The most significant difference was in the need for nipple reconstruction, which was only 18.6% (*n* = 21) in the buried group compared to 51.1% (*n* = 72) in the non-buried group due to the greater proportion of women who underwent NSM (*p* ≤ 0.001). Additional data regarding further reconstructive operations to improve breast cosmesis are presented in Table 4.

## 4. Discussion

This study demonstrates no difference in the rates of flap failure (buried 2.0% vs. non-buried 1.8% *p* = 0.902) or post-operative complications between buried or non-buried free flaps for breast reconstruction. A greater proportion of patients undergoing buried free flap reconstruction completed their breast reconstruction journey in a single stage and had a lower average number of reconstructive procedures (52.2% no further reconstructions in the buried group vs. 25.5% non-buried, *p* < 0.001). Venous flow couplers were utilised to monitor a total of 322 flap, including 153 buried flaps, with no flap losses occurring without being identified in advance via a loss of audible venous flow signal. There was a 2.0% negative takeback rate relating to loss of venous coupler signal but a perfused flap on return to theatre. There was no reported morbidity with coupler wire removal at the bedside.

There were no significant differences in baseline demographic data between the buried and non-buried groups, other than a greater proportion of NSM (buried 57.5% vs. non-buried 7.7%, *p* ≤ 0.001) and immediate reconstructions in the buried group (buried 82.4% vs. non-buried 60.9% *p* ≤ 0.001). Patients who had a mastectomy and subsequent delayed autologous reconstruction will have a deficiency of skin, and therefore have greater representation in the non-buried group due to their skin paddle. This study showed an increased intra-operative time for buried flaps (buried 416.4 min (SD ± 117.9) vs. non-buried 383.7 min (SD ± 287.8) *p* = 0.031). This is likely to reflect the higher proportion of patients undergoing immediate reconstruction in the buried group, as well as the higher proportion of unilateral patients that underwent concurrent symmetrisation (buried 31.9% vs. non-buried 21.3% *p* = 0.085). We recognise the importance of experienced co-surgeons to facilitate simultaneous operating, reducing the operative time and thus optimising patient safety [17]. A recent meta-analysis by Escandón et al. demonstrated the value of co-surgeon operators on operative time for autologous breast reconstruction, although they found surgical complications comparative compared to a single-surgeon approach [18].

The average ischaemia time in the buried flap group was 62.7 min (SD ± 24.7) compared to 69.0 min (SD ± 25.0) in the non-buried group (*p* = 0.036). However, the clinical significance of this statistical difference is unlikely to be of relevance. There was a significantly longer follow-up period in the non-buried flap group (41.4 months (SD ± 29.2)) compared to patients undergoing buried free flap reconstruction (12.8 months (SD ± 11.4). This is likely to reflect the increasing proportion of buried flaps over the course of the series.

The flap failure rate in both the buried and non-buried cohort was comparable to the most recent published report from the UK National Flap Registry, reporting a return to theatre rate of 7% for free flap reconstructions with a flap failure rate of 2.5% nationwide [19,20]. The results of this study are also in line with a large multi-centre international published series citing a 2% rate of total flap loss in 4577 DIEP flaps [21]. This demonstrates that buried free flaps can be reliably performed and monitored. The perceived increased haematoma and flap failure rate described in smaller unmatched series was not observed in our study [14]. As previously described, we have refined our technique for placing and securing the coupler wire, reducing dislodgement and facilitating safe removal.

Buried free flaps, combined with nipple-sparing approaches and concurrent symmetrisation, significantly reduce the requirement for additional procedures following free flap breast surgery. The UK national average for secondary adjustments following free flap breast surgery is 1.3, which is significantly higher than the average for the buried flap group of 0.62 in this study [19]. Another series from high-volume microsurgical units describes an average rate of 3.3 subsequent surgical episodes after their primary reconstructions [22]. In a series by Frey at al., the assumption that buried flaps lead to fewer revisional surgeries was challenged. A total of 221 free flaps were included in their series, although only 50 of those were buried. They described a greater average of revisional procedures in the buried group than that with a skin paddle (buried 0.82 vs. non-buried 0.44). Revisional procedures included lipofilling and contralateral symmetrising surgery, with some patients in the non-buried cohort choosing not to have their skin paddle excised [5].

The most common secondary procedures in our series across both groups was lipofilling, which was performed significantly more in the non-buried setting (buried 30.1% vs. non-buried 48.2% *p* = 0.003). Nipple reconstruction with local flaps was only performed in 18.6% (*n* = 21) in the buried group compared to 51.1% (*n* = 72) in the non-buried group due to the greater proportion of women who underwent NSM (*p* ≤ 0.001).

Halani et al. introduced the concept of reconstructive burnout following mastectomy to describe women who did not complete their reconstruction due to the emotional and physical toll of repeated operative procedures [7]. Reducing the number of additional surgeries through the adoption of single-stage breast reconstruction and the use of buried flaps can reduce the emotional toll of a protracted reconstructive journey. Additionally, some providers are restricting funding for secondary procedures following reconstruction. These economic considerations are additional motivators for minimising the need for secondary surgery following autologous breast reconstruction [19]. Due to the prioritisation of treating primary cancers, there is often a protracted wait for revisional surgery, which is further extenuated by the ongoing COVID-19 backlog of elective surgical care [23].

In our series, all buried flaps were monitored with venous flow couplers only, without monitoring arterial patency with implantable Dopplers. Venous insufficiency is the most common aetiology for flap re-exploration and subsequent free flap failure, and venous flow couplers can identify a lack of flow almost immediately [21,24]. The loss of arterial flow is less sensitive, as there is a well-recognised issue with falsely transmitted Cook–Schwartz signal in the absence of flow [25,26]. Some studies questioned the reliability of flow couplers, with early small studies suggesting increased rates of thrombosis and higher rates of negative explorations [27]. However, this has not been our experience, along with other larger published studies, which have shown an equivocal rate of microsurgical complications as compared to standard couplers [6,13]. It is of note that a 2.0% rate of negative re-exploration was noted in this study, leading to unnecessary morbidity with return to theatre in two patients. Significantly, there were no flaps losses that were not identified in advance by a loss in the venous coupler signal.

There are several technical considerations when introducing buried flaps into a service. Firstly, a close working relationship with breast surgeons is required in order to ensure that, where appropriate, patients are considered for NSM and that the mastectomy is performed in a way to optimise the breast skin envelope. Our preferred approach was either an IMF incision, Wise pattern or Wise pattern with dermal nipple pedicle, depending on the degree of ptosis (Figure 2). In their series of 163 patients, Salibian et al. observed IMF and inverted-T incisions to have a significantly higher risk of mastectomy flap necrosis [28]. Whilst stratification of mastectomy flap complications was not performed as part of our analysis, there was no significant difference between mastectomy flap or nipple necrosis across the buried and non-buried groups. The IMF approach can sometimes restrict access to the 3rd costal cartilage, requiring the 4th costal cartilage to be prepared, which may result in smaller calibre recipient vessels.

Secondly, the introduction of buried flaps and the associated venous flow coupler monitoring has an increased upfront cost. Initially, it requires an investment in monitoring equipment and training of staff. Additionally, flow couplers have a higher unit cost compared to standard venous couplers, calculated as GBP 680 vs. GBP 170, respectively, by Chadwick et al. [13]. However, these incrementally increased costs need to be offset by the reduced need for secondary surgery demonstrated with buried flaps [6,29].

Familiarity with monitoring using flow couplers has additional benefits, even for patients with cutaneous skin paddles. The authors have routinely used flow couplers in addition to skin paddles and cutaneous Doppler monitoring due to the earlier detection of flap compromise. Nursing staff have reported preferring the binary outcome of whether flow is present or not compared to the subjective assessment of skin paddles [11]. A subjective assessment of skin paddles can be challenging in non-Caucasian skin and, as such, is associated with a higher incidence of flap loss due to delayed flap salvage [30].

## 5. Limitations

The limitations of this study include that this series is composed of a single surgeon’s experience and, therefore, may limit its reproducibility. Due to the inclusion of patients in the private sector, with greater consultant lead involvement, this may limit the generalisability of this research to the public setting. Due to the observational nature of this study, we are unable to associate the causality of buried flaps with our lower rate of secondary surgery. It is likely this rate is due to multiple contributing factors, and represents the ethos of a department towards NSM, immediate breast reconstruction and concurrent contralateral symmetrising surgery where appropriate.

Classifying single-stage breast reconstruction as truly ‘single stage’ requires an extended period of follow-up as, over time, patients may seek late revision surgery due to the impact of adjuvant treatment or change over time. We have not adjusted for neo-adjuvant or adjuvant radiotherapy across our series, which—along with other factors such as BMI—may affect the rate of secondary procedures, such as lipofilling. Although adequate, the mean follow-up period in this series is 41.4 months (SD ± 29.2) in patients undergoing non-buried flaps, compared to 12.8 months (SD ± 11.4) in patients undergoing buried free flaps. Therefore, some patients may still require revision surgeries to improve breast aesthetics, which has not been captured by our study.

Whilst leaving the breast with less stigmata of reconstruction—through NSM and buried flaps—subjectively confers a more pleasing aesthetic result, there are no validated patient-reported outcome measures (PROMs) data to support this in our series. Other studies have utilised the Breast-Q to demonstrate a higher psychosocial and sexual wellbeing score in the NSM cohort compared to SSM [8,31]. In addition, whilst it is likely that the reduction in secondary surgery will offset additional costs of venous flow couplers, no formal cost analysis was included in this study. Due to the heterogeneity of costs across differing healthcare settings, including staffing and theatre costs, this would need to be considered on an individualised basis depending on local healthcare economics.

### Summary and Future Directions

Just as autologous reconstruction is offered to every woman undergoing mastectomy regardless of local service provision, we propose that NSM and buried single-stage reconstruction should be offered to all patients where it is oncologically safe. Further larger studies are required to validate the safety of venous flow couplers as their reliability for detecting flap compromise is vital to this technique. Further work on PROMs in both the buried flaps and those with skin paddles would provide an objective indicator of the superiority of buried flaps.

## Figures and Tables

**Table 1:** Patient demographics and operative description.

	Buried		Non-Buried		*p* Value
	*n* (%)	%	*n* (%)	%	
Total patients	113		141		
Total flaps	153	47.5%	169	52.0%	
Age	48.3		49.8		0.337
Co-morbidities					*p* value
Hypertension	10	8.8%	17	12.1%	0.410
BMI	9	8.0%	11	7.8%	0.962
Smoking	4	3.5%	14	9.9%	0.049
Ex smoker	4	3.5%	1	0.7%	0.107
Diabetes	5	4.4%	9	6.4%	0.497
Asthma	9	8.0%	6	4.3%	0.213
Other	11	9.7%	23	16.3%	0.126
Flaps					
DIEP	120	78.4%	143	84.6%	0.129
PAP	27	17.6%	18	10.7%	0.056
SGAP	5	3.2%	5	3.0%	0.890
SIEA	1	0.65%	2	1.2%	0.613
ALT	0	0.0%	1	0.6%	0.337
Unilateral vs. bilateral					
Unilateral	52	34.0%	87	51.5%	0.001
Bilateral	54	35.3%	43	27.2%	0.138
Bilateral: one buried/one non-buried	3	2.0%	3	1.8%	0.915
Unilateral—stacked	16	10.5%	10	5.9%	0.135
Unilateral—bipedicle	20	13.1%	25	14.8%	0.656
Bilateral—stacked	7	4.6%	0	0.0%	0.005
Immediate vs. delayed					
Immediate	124	82.4%	103	60.9%	<0.001
Delayed	29	19.0%	66	39.1%	<0.001
Immediate symmetrisation procedure	28	31.9%	26	21.3%	0.085
Mastectomy					
Nipple sparing	88	57.5%	13	7.7%	<0.001
Skin reducing	46	30.1%	8	4.7%	<0.001
Free nipple graft	9	5.9%	1	0.6%	0.007

BMI—body mass index, DIEP—deep inferior epigastric perforator, PAP—profunda artery perforator, SGAP—superior gluteal artery perforator, SIEA—superficial inferior epigastric artery, ALT—anterolateral thigh.

**Table 2:** Intra-operative details.

	Buried		Non-Buried		*p* Value
	Time (min)	SD	Time (min)	SD	
Ischaemia time	62.6	±24.7	69.0	±24.9	0.036
Flap weight	509.1	±284.8	621.6	±287.8	0.002
Operating time	416.4	±117.9	383.6	±287.8	0.031

**Table 3:** Incidence of complications in buried and non-buried flaps.

	Buried		Non-Buried		*p* Values
	*n*	%	*n*	%	
Flap					
Haematoma	7	4.6%	7	4.1%	0.849
Fat necrosis	2	1.3%	4	2.4%	0.483
Flap loss	3	2.0%	3	1.8%	0.902
Negative takeback	3	2.0%	1	0.6%	0.268
RTT vein	1	0.7%	2	1.2%	0.621
Partial flap loss	0	0%	1	0.6%	0.341
Intra-operative flap issues					
Cephalic turndown	2	1.3%	3	1.8%	0.734
Arterial issue	1	0.7%	0	0%	0.293
SIEV supercharge	2	1.3%	0	0%	0.136
Mastectomy skin/nipple					
Wound—conservation	10	6.5%	11	6.5%	0.992
Wound—re-admission/re-operation	7	4.6%	6	3.6%	0.641
NAC loss	4	2.6%	2	1.2%	0.343
Donor site					
Wound—conservative	4	2.6%	11	6.5%	0.098
Wound—infection	4	2.6%	2	1.2%	0.343
Wound dehiscence—RTT	3	2.0%	6	3.6%	0.388
Seroma	3	2.0%	3	1.8%	0.902
Bulge	0	0%	1	0.6%	0.341

RTT—return to theatre, SIEV—superficial inferior epigastric vein, NAC—nipple areolar complex.

**Table 4:** Additional procedures to improve breast or donor site cosmesis.

		Buried		Non-Buried		*p*
Additional Procedures		*n*	%	*n*	%	
	Lipofilling	34	30.1%	68	48.2%	0.003
	Nipple reconstruction	21	18.6%	72	51.1%	<0.001
	Scar revision	7	6.2%	7	5.0%	0.669
	Symmetrisation	15	13.3%	23	16.3%	0.500
	Dog ear excision	2	1.8%	5	3.5%	0.390
	FTSG to mastectomy wound	2	1.8%	0	0%	0.113
Total additional procedures for breast appearance						
	No further procedures	59	52.2%	36	25.5%	<0.001
	1	44	38.9%	72	51.1%	
	2	6	5.3%	19	13.5%	
	3	3	2.7%	5	3.5%	
	>3	1	0.9%	5	3.5%	
	Average	0.62		1	1.12	<0.001

FTSG—full-thickness skin graft.

## Data Availability

The data supporting the findings of the study are available upon request from the corresponding author (H.C.).
